# MyμAlbumin: A Cutting-Edge Immunoturbidity-Based Device with Real-Time and Seamless Data Transmission for Early Detection of Chronic Kidney Disease at the Point of Care

**DOI:** 10.3390/bios15060391

**Published:** 2025-06-17

**Authors:** Wanna Chaijaroenkul, Napaporn Youngvises, Artitaya Thiengsusuk, Tullayakorn Plengsuriyakarn, Jakkrapong Suwanboriboon, Kridsada Sirisabhabhorn, Wanchai Meesiri, Kesara Na-Bangchang

**Affiliations:** 1 Graduate Program in Bioclinical Sciences, Chulabhorn International College of Medicine, Thammasat University, Pathum Thani 12120, Thailand; cwanna@tu.ac.th (W.C.); tulpleng@tu.ac.th (T.P.); 2 Drug Discovery and Development Center, Office of Advanced Science and Technology, Thammasat University, Pathum Thani 12120, Thailand; napaporn@tu.ac.th (N.Y.); artitaya@tu.ac.th (A.T.); 3 Bangkok High Lab Co., Ltd., Bang Khen District, Bangkok 10220, Thailand; js_aek@hotmail.com (J.S.); wanchai.meesiri@bangkokhighlab.com (W.M.); 4 Department of Medical Technology Laboratory, Thammasat University Hospital, Pathum Thani 12120, Thailand; kridsirimttu@gmail.com

**Keywords:** microalbumin, point-of-care (PoC) medical device, chronic kidney disease

## Abstract

Microalbuminemia, characterized by a urinary albumin concentration between 20 and 200 mg/L, is a critical marker in assessing the risk of chronic kidney disease (CKD), diabetic nephropathy, and various other chronic conditions. Previously, we developed and validated the MyACR point-of-care (PoC) device, which facilitates the monitoring of CKD progression through real-time data transmission, thus enhancing patient management. This device utilizes a spectrophotometric dye-binding assay to measure albumin and creatinine concentrations in urine samples, providing an albumin-to-creatinine ratio (ACR) result. In the present study, we introduced a refined version of the PoC device, MyμAlbumin, designed to offer a simple, accurate, specific, sensitive, and rapid method for detecting microalbumin in urine as an early indicator of CKD and related diseases. The measurement is based on a specific immunoturbidimetric assay in a microcuvette, using a total solution volume of 125 µL (n = 5 for each validation test). The MyμAlbumin device demonstrated excellent performance, achieving high accuracy (%DMV ≤ 4.67) and precision (%CV < 5) and a strong correlation (R^2^ > 0.995) with laboratory spectrophotometry (dye-binding assay) and reference hospital-based immunoturbidimetric assay. Its high sensitivity (LOQ = 5 mg/L) positions MyμAlbumin as a highly viable and cost-effective tool for clinical use. Additionally, the device supports real-time, seamless data transmission, making it ideal for integration into remote healthcare settings.

## 1. Introduction

Microalbuminuria refers to the presence of small amounts of albumin (20–200 mg/L, or an albumin-to-creatinine ratio (ACR) of 30–300 mg/g creatinine) in the urine, which is typically undetectable under normal circumstances [1]. Microalbumin is a crucial marker in the early detection of kidney damage and is closely linked to a range of chronic conditions. By identifying microalbumin in urine, clinicians can assess the risk of chronic kidney disease (CKD), diabetic nephropathy, hypertension-related kidney damage, cardiovascular diseases, obesity-associated kidney dysfunction, and autoimmune kidney disorders [1,2,3,4,5]. Regularly monitoring urinary microalbumin levels is vital in managing these conditions and preventing their progression to more severe health complications.

Point-of-care (PoC) devices for microalbumin testing are transforming the diagnosis and monitoring of kidney diseases. These devices enable the rapid, on-site assessment of microalbuminuria [6,7,8], offering a fast and efficient way to track kidney health. Technological advancements have made PoC devices essential to clinical care, providing quick results that facilitate timely medical interventions. The integration of seamless data transmission into these devices represents a significant breakthrough in chronic kidney disease (CKD) management, enabling rapid and accurate assessments, improving patient engagement, and enhancing healthcare outcomes through early interventions. Their portability and cost-effectiveness also make them particularly valuable in low-resource settings.

Our research group previously developed and validated the MyACR point-of-care (PoC) device to facilitate the monitoring of chronic kidney disease (CKD) progression, incorporating real-time data transmission to enhance patient management [9]. By combining the urinary albumin-to-creatinine ratio (ACR) with the estimated glomerular filtration rate (eGFR), the CKD prognosis can be further refined [10]. A urinary ACR greater than 300 mg/g creatinine is classified as ‘macroalbuminuria’, indicating significant kidney damage, and is often associated with more advanced stages of CKD [1,2]. The MyACR device utilizes spectrophotometric dye-binding and colorimetric Jaffe assays to measure albumin and creatinine concentrations in urine samples, thereby providing accurate uACR analysis. The device demonstrated 100% sensitivity, specificity, accuracy, positive predictive value (PPV), and negative predictive value (NPV) in detecting severe nephropathy (uACR > 300 mg/g creatinine), showing a strong correlation with reference methods (R^2^ = 0.9720 − 0.9836) and a high agreement rate (96.11%) in Bland–Altman analysis.

In the present study, we further developed MyμAlbumin, a simple, accurate, sensitive, and rapid PoC device designed to measure urine microalbumin as an early indicator of CKD and associated diseases. This measurement is based on a specific immunoturbidimetric assay.

## 2. Materials and Methods

### 2.1. Chemicals and Reagents

Anti-albumin antibodies produced in goats were purchased from Sigma-Aldrich (St. Louis, MO, USA). Phosphate-buffered saline (PBS) at pH 7.4 and polyethylene glycol 6000 (PEG) were obtained from Power Tech Chemical (Bangkok, Thailand). Human serum albumin (HSA) was purchased from Sisco Research Laboratories (Mumbai, India).

A stock solution of HSA (2 mg/mL) was prepared using PBS and stored at 4 °C. Working solutions were prepared by diluting the stock solution with PBS before use. PEG (4%, *w*/*v*) was prepared by dissolving 40 g of PEG in 1 L of PBS. Artificial urine was prepared following the method described in a previous publication [11]. The anti-albumin antibody was diluted 1:5 with buffered PEG before use.

### 2.2. Instrumentation and Measurement Platform

**Figure 1:**
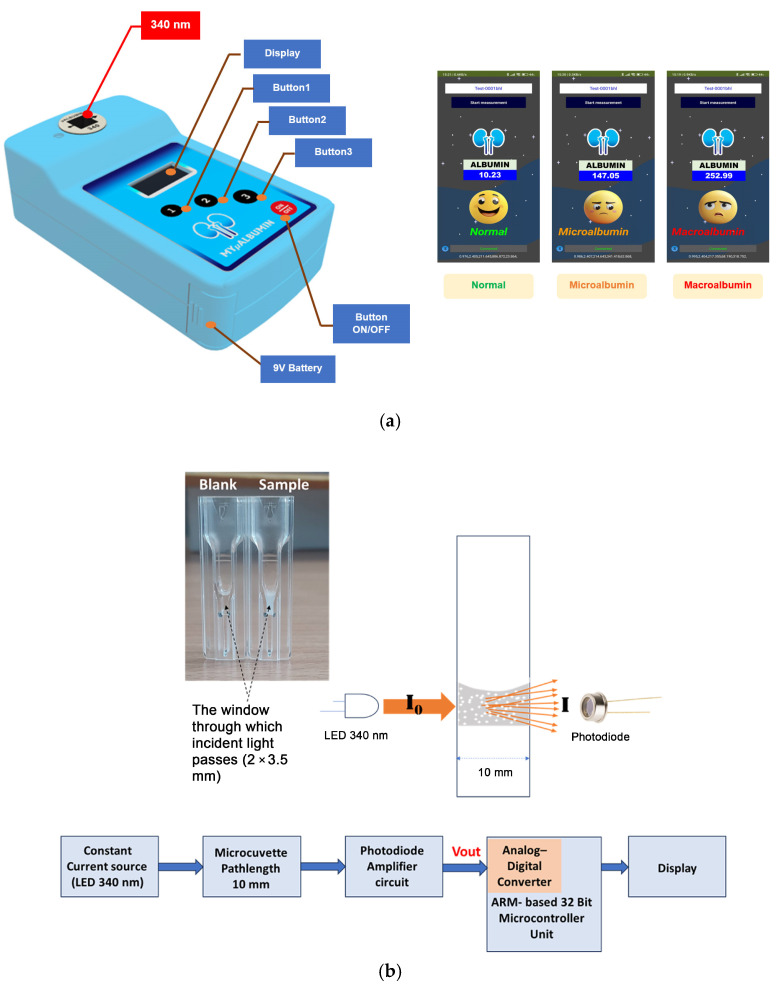
(**a**) MyμAlbumin device and (**b**) measurement platform.

**Figure 2:**
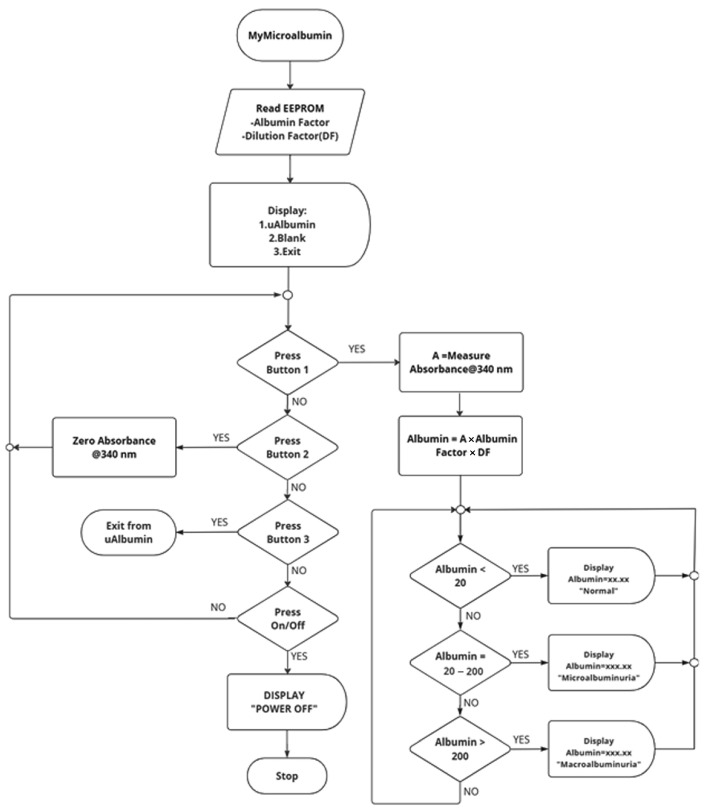
Flowchart showing the operational workflow of MyμAlbumin.

The MyμAlbumin device is a turbidity-based spectroscopic PoC testing system developed to quantify urinary albumin levels. The instrumentation integrates an optical sensing platform controlled by a microcontroller unit (MCU), which regulates a constant current source to emit ultraviolet (UV) light at a wavelength of 340 nm. This light passes through a cuvette positioned in the optical path and is subsequently detected by a photodiode amplifier circuit. The resulting output signal (Vout) is digitized via an analog-to-digital converter (ADC) and processed using a signal filtering protocol to eliminate noise and irrelevant frequencies. To minimize reagent consumption—particularly due to the high cost of antibodies—a disposable UV-transparent microcuvette (15 mm center height; BRAND^®^, Wertheim, Germany) was utilized. As shown in Figure 1, the microcuvette shares the external dimensions of a standard cuvette (12.5 × 12.5 × 45 mm) with a 10 mm optical pathlength but requires a significantly reduced sample volume due to a minimized detection window (2 × 3.5 mm). For the experiments conducted in this study, the total sample volume required per test was 125 μL. Figure 1 illustrates the physical test platform, while Figure 2 provides a schematic overview of the device’s operational flow, detailing the electronic signal generation, light transmission, and detection processes employed throughout the measurement procedure.

Upon mixing albumin with the specific antibody within the microcuvette, the solution exhibited increased turbidity due to the formation of immune complexes. When ultraviolet (UV) light at 340 nm (I_0_), emitted from the LED source, was transmitted through the turbid solution, light scattering by suspended particles occurred. The 340 nm emission wavelength was chosen due to its spectral specificity and stability which are suitable for immunoturbidimetric assay. This scattering effect reduced the intensity of the transmitted light (I) received by the photodiode detector. The LED is driven by a constant current source regulated by the MCU, ensuring stable optical output with power fluctuations limited to within ±1% during measurement. To maintain measurement consistency over time and compensate for potential LED aging or environmental effects, the system incorporates an automatic calibration routine before each measurement cycle. This routine measures the baseline intensity of the LED light without the sample (blank measurement), allowing subsequent absorbance calculations to be normalized against the reference intensity. The transmitted light passing through the sample is detected by a photodiode, which converts the optical signal to an electrical voltage. This voltage is amplified and digitized using a 12-bit ADC embedded in the MCU, providing 4096 discrete quantization levels. This resolution is sufficient to detect subtle changes in light intensity related to varying albumin concentrations in urine. To improve signal quality, the digital data from the ADC is processed with a low-pass digital filter, specifically a finite impulse response (FIR) filter, configured with a cutoff frequency below 10 Hz to effectively remove high-frequency noise without compromising the integrity of the absorbance signal.

The absorbance (A), which correlates with the concentration of albumin in the sample, was calculated based on the reduction in light intensity according to Equation (1):

A = −log_10_ (I/I_0_) (1)

The albumin concentration was determined by measuring the turbidity in an absorbance mode, with greater cloudiness corresponding to higher absorbance (Figure 1b).

A ∝ Turbidity ∝ x(2)

I = transmitted light, I_0_ = incident light, A = absorbance, x = concentration of albumin (mg L^−1^).

A = ax^2^ + bx + c (3)

Preliminary experimental data demonstrated that the relationship between absorbance (A) and albumin concentration (x) followed a second-degree polynomial model, as represented in Equation (3). The concentration of albumin was automatically calculated by the device’s embedded algorithm, as outlined in the flowchart presented in Figure 2. Based on the computed albumin concentration, the results were categorized and displayed on the device interface according to the following clinical interpretation criteria: (i) **normal** for urinary albumin levels < 20 mg/L, (ii) **microalbuminuria** for levels ranging from 20 to 200 mg/L, and (iii) **macroalbuminuria** for levels > 200 mg/L [12,13].

### 2.3. Measurement of Albumin in Urine Samples Using MyμAlbumin

The quantification of albumin in urine samples using the MyμAlbumin device was performed via an immunoturbidimetric assay, which relies on specific antigen–antibody interactions. In this system, anti-albumin antibodies selectively bind to albumin present in both artificial and clinical urine samples, leading to the formation of immune complexes. These complexes induce turbidity, and the resulting increase in light scattering is directly proportional to the albumin concentration in the sample [14]. To enhance the stability and uniformity of the immune complexes, 4% PEG was added to the reaction mixture.

Prior to measurement, clinical urine samples were allowed to settle at room temperature (25 °C) for 15 min to facilitate sedimentation of blood components and cellular debris that could interfere with absorbance readings. The reaction mixture prepared in the microcuvette consisted of 10 μL of a urine sample or albumin standard, 15 μL of phosphate-buffered saline (PBS), and 100 μL of an anti-albumin antibody solution (1:5 dilution in PBS). After mixing, the reaction was incubated, and the absorbance measured at 340 nm using the MyμAlbumin device.

To optimize assay performance, critical reaction parameters were systematically evaluated, including temperature, incubation time, and saline concentration.

Temperature: The assay was tested over a temperature range of 20 °C to 40 °C. The optimal incubation temperature was selected as 37 °C because it mimics physiological conditions, promotes efficient antigen–antibody binding, and ensures the stability of immune complexes without increasing non-specific interactions.

Incubation time: Incubation periods from 5 to 30 min were assessed to determine the optimal balance between assay speed and signal stability. Fifteen minutes was chosen as the final incubation time because it allowed sufficient time for complete and consistent immune complex formation while maintaining high throughput and minimizing total assay time. Shorter incubation times (e.g., 5–10 min) resulted in lower turbidity signals and poor reproducibility, while longer times (e.g., >20 min) did not significantly improve the signal and introduced variability due to temperature sensitivity and evaporation.

Saline concentration: The PBS buffer’s NaCl concentration was varied between 0.5% and 1.5%. A concentration of 0.9% NaCl (physiological saline) produced the most favorable results, supporting optimal protein interactions with minimal non-specific binding and background interference.

To address potential analytical errors at high albumin concentrations, particularly the “hook effect”—a phenomenon in which excess antigen prevents effective cross-linking with antibodies—the assay was specifically evaluated for performance at supraphysiological albumin levels (>300 mg/L). Serial dilutions of high-concentration albumin standards were used to define the upper dynamic range of the assay. The system was programmed to automatically flag any sample with absorbance values outside the linear detection range and prompt the user to dilute the sample and remeasure it. This approach ensures that samples exceeding the upper threshold do not produce underestimated values due to antigen excess.

The final optimized assay conditions, 37 °C incubation for 15 min in 0.9% NaCl buffer, were applied to all subsequent analyses. Under these conditions, absorbance values were measured and processed through the device’s integrated software, which employed a second-degree polynomial calibration curve to calculate urinary albumin concentration. These measures collectively ensured robust, reproducible, and clinically reliable performance of the MyμAlbumin system, while minimizing errors due to phenomena such as the hook effect.

### 2.4. Validation of Test Performance of MyMicroalbumin

#### 2.4.1. Calibration Curves

Calibration curves for albumin detection in artificial urine were constructed by repeatedly analyzing samples containing a range of albumin concentrations (0 to 500 mg/L). The analysis followed the previously outlined procedure.

#### 2.4.2. Accuracy

The analytical accuracy of the MyµAlbumin method for quantifying albumin in artificial urine was assessed by evaluating both intra-day (repeatability) and inter-day (reproducibility) performance. This was conducted through repeated measurements of five sample sets spiked with albumin at three concentration levels (20, 100, and 300 mg/L). Accuracy was calculated as the percentage deviation of the measured mean values from the nominal (% deviation from the mean value: %DMV) concentrations determined by a standard laboratory spectrophotometer, using the following formula:

Accuracy (%) = [(Measured mean − Nominal value)/Nominal value] × 100(4)

#### 2.4.3. Precision

The precision of the MyµAlbumin assay for detecting albumin in artificial urine was evaluated by examining intra-day (repeatability) and inter-day (reproducibility) variability. This was conducted through repeated testing of five sets of samples, each spiked with albumin at concentrations of 20, 100, and 300 mg/L. Precision was expressed as the coefficient of variation (CV), calculated by dividing the standard deviation by the mean and multiplying by 100:

%CV = (Standard Deviation/Mean) × 100(5)

#### 2.4.4. Specificity

The specificity of the test was evaluated by comparing albumin concentrations in clinical urine samples (n = 25) measured using MyμAlbumin after sedimentation (Section 2.3) with those measured in clinical urine samples without sedimentation using laboratory spectrophotometry and hospital-based immunoturbidimetric methods.

#### 2.4.5. Limit of Quantification

The limit of quantification (LOQ) for the MyµAlbumin assay was defined as the lowest concentration of albumin in spiked artificial urine samples that yielded a coefficient of variation (CV) within ±20% of the target concentration.

### 2.5. Application of MyμAlbumin to Clinical Samples

This study received ethical approval from the Ethics Committee of the Faculty of Medicine, Thammasat University (Project No. MTU-EC-OO-2-1-168/66; Approval No. 266/2566). It was conducted at Thammasat Chalermprakiet Hospital and the Thammasat University Center of Excellence in Pharmacology and Molecular Biology of Malaria and Cholangiocarcinoma, in accordance with the principles of the Declaration of Helsinki. All participants provided written informed consent prior to enrollment.

Random urine samples (2–3 mL) were collected from 25 patients who presented at the Outpatient Department. All samples were stored at −20 °C until analysis (within 1 day). Samples were left at room temperature for 10 min. The albumin concentration of each sample was determined using the MyμAlbumin device and compared with laboratory spectrophotometry and hospital-based immunoturbidimetric methods. The specificity of the assay was evaluated in all urine samples as described in Section 2.4.4. The measurement of albumin concentration using both MyμAlbumin and the reference methods was based on a turbidimetric inhibition immunoassay.

### 2.6. Statistical Analysis

Statistical analyses were conducted using SPSS version 22.0. Quantitative data are presented as median values along with their respective ranges. The percentage deviation from the mean value (%DMV) was calculated to compare urinary microalbumin concentrations obtained from the MyµAlbumin device with those measured by a laboratory spectrophotometer (SPECTROstar Nano microplate reader, BMG LABTECH, Ortenberg, Germany) and a hospital-based immunoturbidimetric method. Correlations between the MyµAlbumin results and the reference methods were assessed using Spearman’s rank correlation test, with statistical significance set at α = 0.05.

## 3. Results

### 3.1. Validation of Test Performance of MyMicroAlbumin Device

#### 3.1.1. Calibration Curves

**Figure 3:**
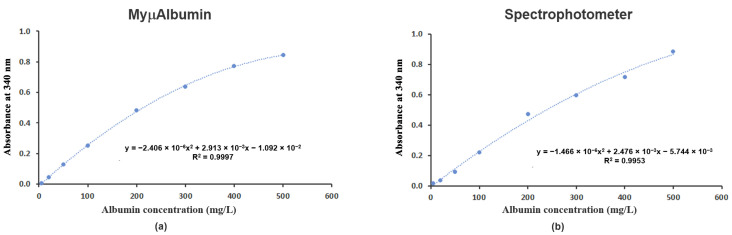
Calibration curves of albumin in artificial urine at the concentration range of 5–500 mg/L determined using (**a**) MyμAlbumin and (**b**) the well-plate spectrophotometer.

Albumin concentrations in artificial urine were analyzed using both the MyµAlbumin device and a well-plate spectrophotometer (SPECTROstar Nano microplate reader; BMG LABTECH, Ortenberg, Germany), with calibration performed over a concentration range of 0–500 mg/L. Non-linear calibration curves were fitted to the data and are represented by the polynomial Equations (6) and (7), respectively (Figure 3a,b).

A = −2.406 × 10^−6^x^2^ + 2.913 × 10^−3^x − 1.092 × 10^−2^ with R^2^ = 0.9995 (6)

A = −1.466 × 10^−6^x^2^ + 2.476 × 10^−3^x − 5.744 × 10^−3^ with R^2^ = 0.9953 (7)

#### 3.1.2. Accuracy

MyµAlbumin demonstrated good accuracy in both intra-day and inter-day analyses for albumin quantification in artificial urine, as evidenced by minimal deviations between measured and theoretical values (actual spiked concentrations). When compared to results from a laboratory spectrophotometer and a hospital-based immunoturbidimetric assay, the percentage deviation from the mean (%DMV) remained below 4.67% across all tested concentrations (Table 1).

#### 3.1.3. Precision

Acceptable levels of intra- and inter-assay variation were observed in albumin measurements using MyµAlbumin, with coefficients of variation (CV) not exceeding 11.01% across all tested concentrations. These results were comparable to those obtained using laboratory spectrophotometry and hospital-based immunoturbidimetric methods (Table 1).

#### 3.1.4. Specificity

Albumin concentrations in clinical urine samples (n = 3) measured using MyμAlbumin after sedimentation (Section 2.3) compared with those measured in clinical urine samples without sedimentation using the laboratory spectrophotometer and hospital-based immunoturbidimetric methods were within an acceptable accuracy range, with an average median %DMV of <5%.

#### 3.1.5. Limit of Quantification

The LOQ of albumin in urine determined using MyμAlbumin and the laboratory spectrophotometer with %CV ≤ 20% was 5 mg/L, using 10 µL urine samples.

### 3.2. Application to Clinical Samples

**Figure 4:**
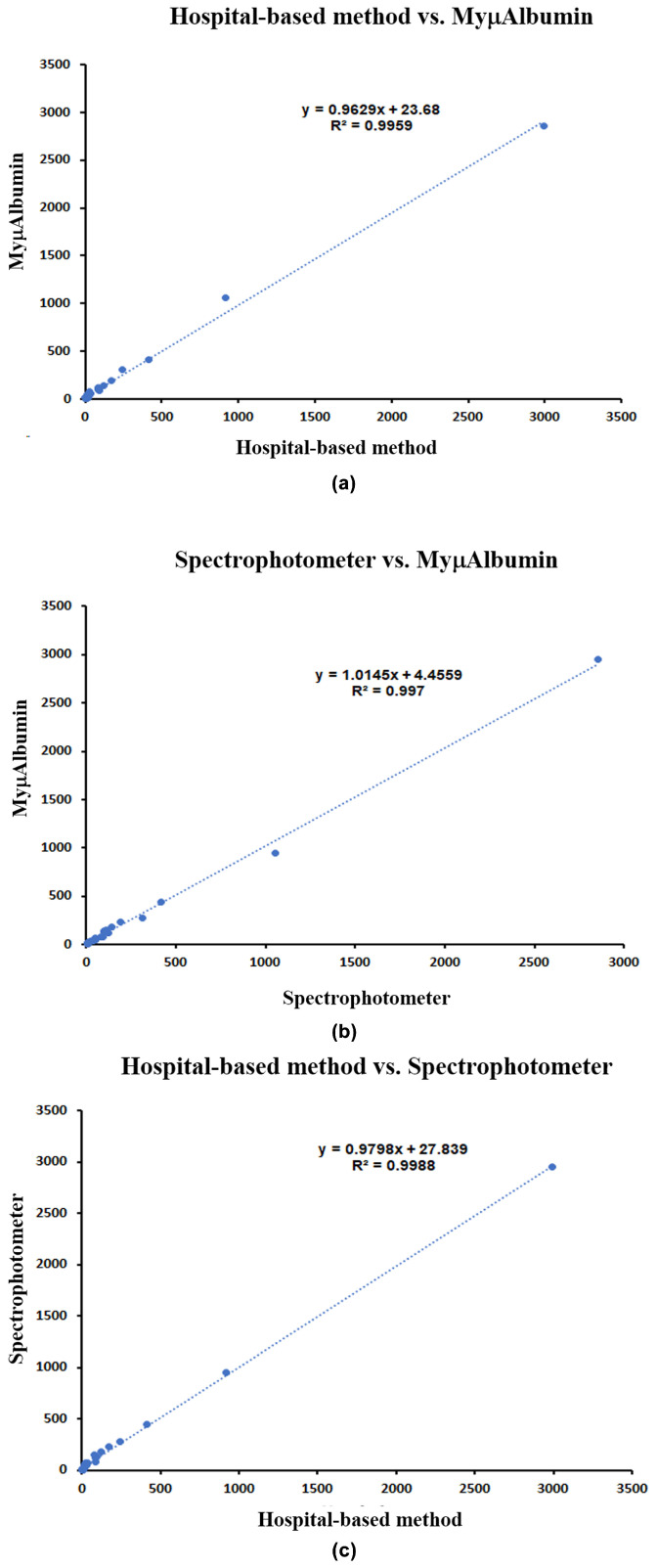
Correlation between urinary albumin concentrations from 25 subjects measured using (**a**) MyμAlbumin vs. hospital-based immunoturbidimetric methods; (**b**) MyμAlbumin vs. laboratory spectrophotometer; and (**c**) laboratory spectrophotometer vs. hospital-based immunoturbidimetric methods.

To assess the clinical utility of MyµAlbumin, albumin levels in urine samples from 25 patients were measured using the device and compared to values obtained from a laboratory spectrophotometer and a hospital-based immunoturbidimetric assay. The median percentage deviation from the mean (%DMV) was 1.45% (range: −34.05 to 12.29) for the spectrophotometer and 1.58% (range: −37.50 to 35.10) for the immunoturbidimetric method. A strong correlation between the two reference methods was observed, with an R^2^ value of 0.995 or higher (Figure 4a–c).

## 4. Discussion

Microalbuminuria, defined as a urinary albumin concentration between 20 and 200 mg/L, serves as an early marker of kidney damage and a critical predictor of chronic kidney disease (CKD), diabetic nephropathy, and cardiovascular diseases [15]. Therefore, precise and sensitive detection methods are crucial for achieving early diagnosis, enabling effective disease management. Traditional techniques for measuring urinary albumin include immunoassays, spectrophotometry, chromatography-based methods, and point-of-care (PoC) devices. High-performance liquid chromatography (HPLC) is regarded as the gold standard for albumin quantification due to its high precision and specificity, which enables the detection of sub-microgram levels [16]. This method separates albumin molecules based on molecular interactions with a stationary phase. However, it is not suitable for PoC applications due to the requirement for expensive equipment and skilled personnel [17]. Dipstick tests are the most commonly used rapid screening tool for microalbuminuria. They detect albumin through colorimetric changes when exposed to urine. Despite their wide availability, these tests have limitations, including poor sensitivity (LOQ of 30 mg/L), high variability, and susceptibility to false positives or negatives due to fluctuations in urine pH and interference from other proteins [18]. Furthermore, they lack quantitative accuracy and cannot replace laboratory-based confirmatory tests [19]. Spectrophotometry is another widely used method due to its affordability and simplicity. This technique measures albumin concentration by monitoring the binding of albumin to dyes such as bromocresol green or pyrogallol red, which results in a change in absorbance at specific wavelengths [19]. While spectrophotometry offers good analytical precision (CV < 10%), it can lack specificity due to interference from other substances in urine [20]. Immunoassays, including turbidimetric and nephelometric methods, detect albumin through antigen–antibody interactions, measuring changes in light scattering or absorbance [21]. These assays offer high sensitivity (LOQ 2–5 mg/L) and specificity, but issues such as batch-to-batch variability and reagent instability can affect measurement reliability [19]. Furthermore, despite their accuracy, immunoassays are expensive and require specialized equipment and trained personnel, making them unsuitable for rapid, decentralized testing [22].

In the present study, the newly developed point-of-care (PoC) device MyμAlbumin was validated for the determination of albumin concentrations in urine. The calibration curves exhibited a polynomial relationship with high correlation coefficients (R^2^ ≥ 0.995), indicating that the device accurately detects albumin levels across a broad range (5–500 mg/L). Unlike traditional linear regression, the calibration curves were fitted with polymodal regression, ensuring precise alignment with the measured data. The strong correlation with the laboratory spectrophotometer further supports the reliability of MyμAlbumin [19]. The device’s intra- and inter-day accuracy (%DMV) remained within acceptable limits (≤4.67%), suggesting minimal deviation between measured and expected values, which affirms its consistency. Precision was assessed through intra- and inter-assay variation, with MyμAlbumin maintaining a coefficient of variation (%CV) ≤ 11.01% across all concentrations. The device demonstrated superior precision at lower albumin concentrations (e.g., 20 mg/L), with lower %CV values than the laboratory spectrophotometer (1.97% vs. 9.81% intra-day, 1.30% vs. 11.01% inter-day). This indicates that MyμAlbumin provides greater consistency at lower concentrations, a clinically significant feature for the early detection of albuminuria [19]. Both MyμAlbumin and the laboratory spectrophotometer exhibited an LOQ of 5 mg/L using 10 µL urine samples, confirming the device’s sensitivity for clinical applications. MyμAlbumin offers a balanced combination of accuracy, precision, and clinical usability, making it superior to dipstick tests and comparable to spectrophotometry for the detection of urine albumin. Additionally, MyμAlbumin presents a more straightforward and efficient PoC solution than laboratory-based spectrophotometry, especially in settings where rapid intervention is crucial [18,19].

When applied to 25 clinical urine samples, the portable MyμAlbumin device strongly correlated with the laboratory spectrophotometer (R^2^ ≥ 0.995), suggesting its clinical utility and reliability. The accuracy (%DVM) of MyμAlbumin compared to the hospital-based immunoturbimetric assay was within 5%, highlighting its consistent performance in clinical settings. The potential influence of non-specific binding and matrix effects from urinary metabolites on albumin detection is an important consideration in the development of the MyμAlbumin assay. Although 340 nm was chosen as the excitation wavelength due to its compatibility with the immunoturbidimetric reaction and the optical properties of the immune complexes, it is recognized that some urinary components may absorb or scatter light at this wavelength, potentially causing interference. To minimize such effects, clinical urine samples were allowed to settle at room temperature for 15 min before measurement, enabling the sedimentation of blood cells, cell debris, and larger particulates that could contribute to turbidity unrelated to albumin–antibody complexes. Additionally, the assay utilizes highly specific anti-albumin antibodies that bind selectively to albumin molecules, thereby reducing the likelihood of non-specific complex formation with other urinary proteins or metabolites. Calibration curves were generated using both albumin standards prepared in buffer and albumin spiked into artificial urine matrices to account for background absorbance and scattering effects inherent to urine composition. The device’s software measures a baseline absorbance signal before sample addition, allowing the subtraction of background absorbance from the final measurement, thus compensating for non-specific optical interference at 340 nm. Despite these measures, minor matrix effects cannot be entirely ruled out, and ongoing validation includes testing samples with known interfering substances to assess assay specificity. Future iterations may explore alternative wavelengths or dual-wavelength correction to further improve robustness against complex urine matrices.

Commercially available PoC devices for albumin detection include the Abbott Afinion 2 Analyzer (immunoassay-based) [23], Siemens DCA Vantage Analyzer (immunoassay-based) [6,24], QuikRead Go Analyzer (immunoturbidimetric) [25], SURESIGN FINECARE Analyzer (immunoassay-based) [26,27], and Siemens CLINITEK Microalbumin 2 (urine dipstick test) [28] (Table 2). The performance of MyμAlbumin is generally comparable to other commercially available PoC devices in terms of accuracy, precision, sensitivity, sample volume, and turnaround time. The sensitivity of MyμAlbumin is similar to other immunoassay-based PoC devices. However, the device offers several advantages, including its cost-effectiveness (USD 500 for the device and USD 5 per test), a broader calibration range (0–500 mg/L), and real-time seamless data transmission, which enhances patient management and facilitates timely interventions.

## 5. Conclusions

The PoC device MyμAlbumin demonstrated high accuracy and precision and a strong correlation with laboratory spectrophotometry and hospital-based immunoturbidity assays for urine albumin measurement. Its high sensitivity (LOQ = 5 mg/L) makes it a viable option for clinical use. The robustness of the device for clinical application as a tool for immediate screening in individuals at risk of CKD and related health conditions with microalbuminuria requires further validation.

## 6. Patents

The innovation patent ‘MyμAlbumin’ has been approved by the Department of Intellectual Property of Thailand (No. 2503001204).

## Figures and Tables

**Table 1:** Accuracy and precision (intra- and inter-day) for the determination of albumin in artificial urine using MyμAlbumin compared with the laboratory spectrophotometer.

	Albumin Concentration (mg/L)	Precision (%CV)		Accuracy (%DMV)	
		Intra-Day	Inter-Day	Intra-Day	Inter-Day
MyμAlbumin	20	1.97	1.30	0.03	−1.73
	100	0.33	0.18	4.28	4.28
	300	0.09	0.08	−4.82	−0.97
Spectrophotometer	20	9.81	11.01	−5.02	0.65
	100	4.79	3.91	4.67	−0.77
	300	3.86	8.10	1.33	0.01

**Table 2:** Test performance of MyμAlbumin compared with commercially available PoC device.

Metrics	MyμAlbumin	Abbott Afinion 2	Siemens DCA Vantage	QuikRead Go	Siemens CLINITEK	Suresign FINECARE
**Measurement**	Albumin	Albumin, ACR	Albumin, ACR	Albumin, ACR	Albumin, ACR	Albumin, ACR
**Assay principle**	Immunoturbidimetric	Colorimetric immunoassay	Immunoturbidimetric assay	Immunoturbidimetric assay	Dipstick dye-binding assay	Immunoturbidimetric assay
**Size (inches) and weight (kg)**	6 × 3.5 × 1.5 0.25	10 × 8 × 5 1–1.5	12 × 10 × 6 1.5–2	10 × 8 × 6 1.5–2	6.5 × 9.4 × 6 1–1.5	10 × 8 × 5 1.5–2
**Calibration range (mg/L)**	0–500	5–200	3–300	5–150	10–300	5–200
**Accuracy (%DMV)**	≤4.67%	~3–5%	~4–6%	5–7%	6–8%	3–6%
**Precision (%CV)**	<5	<5	<5	<5	2–10	<5
**Limit of quantification (mg/L)**	5	3	3	3	10	5
**Urine sample volume (μL)**	10	3–10	4–10	10	>5000	5
**Run time (min)**	5–10	5–8	6–7	4	1	6–8

## Data Availability

Data are contained within the article.
